# *Leucheria cantillanensis* (Nassauvieae, Asteraceae), a new species endemic to Central Chile

**DOI:** 10.3897/phytokeys.169.57532

**Published:** 2020-12-08

**Authors:** Nicolás Lavandero, Benito Rosende, María Fernanda Pérez

**Affiliations:** 1 Departamento de Ecología, Facultad de Ciencias Biológicas, Pontificia Universidad Católica de Chile, Avenida Libertador B. O’Higgins 340, Santiago, Chile Pontificia Universidad Católica de Chile Santiago Chile; 2 Instituto de Ecología y Biodiversidad (IEB), Universidad de Chile, Las Palmeras 3425, Santiago, Chile Pontificia Universidad Católica de Chile Santiago Chile

**Keywords:** Asteraceae, Cantillana, *
Leucheria
*, rupicolous flora, taxonomy

## Abstract

A new species, *Leucheria cantillanensis***sp. nov.**, endemic to the coastal mountain range of Central Chile, is described. By using both nDNA and cpDNA, phylogenetic relationships of the new species were investigated. This new species belongs to the acaulescent/subacaulescent clade of *Leucheria*, which is congruent with the morphology of the species. A detailed description, distribution map, insights about its habitat, conservation status, and illustrations are provided. An updated key for acaulescent/subacaulescent species of *Leucheria* from Central Chile is also given.

## Introduction

The ecosystems found in central and southern Chile are one of 35 world biodiversity hotspots, owing to their combination of great diversity and high levels of endemism, and a past and ongoing loss of habitat and biodiversity (Myers et al. 2000; Mittermeier et al. 2005). Central Chile features a Mediterranean-type climate, although important climatic heterogeneity can be found due to latitudinal and altitudinal gradients (Armesto et al. 2007). This heterogeneity, accompanied by the climatic history throughout the Quaternary, must have contributed to the increased species richness and endemism in the area (Arroyo et al. 1995; Villagrán 1995). Many genera show a high number of species within this area, such as *Adesmia* DC., *Chaetanthera* Ruiz & Pav., *Mutisia* L.f., *Oriastrum* Poepp., *Senecio* L., and *Leucheria* Lag., occurring in all available environments, from the coastal dunes, sclerophyllous forests and matorral to Andean vegetation near 4000 m above sea level (a.s.l.).

The genus *Leucheria* comprises 49 species (Crisci 1976; Katinas et al. 2008; Katinas et al. 2018; Jara-Arancio et al. 2019) distributed in Peru, Bolivia, Chile, and Argentina on the continent, plus the Falkland Islands. Most species are concentrated within the Patagonian-Andean and the Subantarctic phytogeographic domains (Cabrera and Willink 1973). The region of Chile with the greatest diversity of *Leucheria* overlaps with the Central and southern Chile biodiversity hotspot, with most species richness and endemism occurring within this area (Moreira-Muñoz et al. 2012). The available specimen coverage of *Leucheria* in Chile is reasonably good, with more than 1200 collected specimens distributed between the most important herbaria in the country, SGO and CONC. However, their collection localities are clustered mainly on accessible regions and along main highways, such as border crossings between Chile and Argentina and Bolivia, ski centres, or the Pan American Route 5 in the North of Chile. Furthermore, many species are poorly collected (e.g. *Leucheria apiifolia* Phil., *Leucheria glabriuscula* Reiche, *Leucheria graui* Katinas, M.C. Tellería & Crisci), and many other species have important geographic gaps among their collections (e.g. *Leucheria achilleifolia* Hook. & Arn., *Leucheria polyclados* (J. Remy) Reiche). Since the most comprehensive revision of the genus (Crisci, 1976), at least two new species (Katinas et al. 2008; Katinas et al. 2018) and a new variety (Ratto et al. 2014), which was later elevated to species level (Jara-Arancio et al. 2019), have been described.

In the context of the ongoing taxonomic revision of the genus, unusual specimens of *Leucheria* were collected at the Reserva Natural Altos de Cantillana (MMA 2018). The locality is known as one of the 72 priority sites for the conservation of biodiversity in Chile and the top in priority within the Metropolitan Region (CONAMA 2004). Altos de Cantillana is located in the coastal mountain range of the Metropolitan Region of Chile, a paradoxically species-rich area with high levels of endemism, but poorly collected and with only a few updated floristic catalogues (Romero and Teillier 2009; García 2010; Flores-Toro and Amigo 2013; Romero-Gárate and Teillier 2014). This work aims to describe a new species of *Leucheria* and investigate its phylogenetic affinities based on molecular data. We also provide a distribution map as well as information on its habitat and phenology, and a provisional assessment of its conservation status.

## Methods

### Herbarium and fieldwork

**Figure 1.**
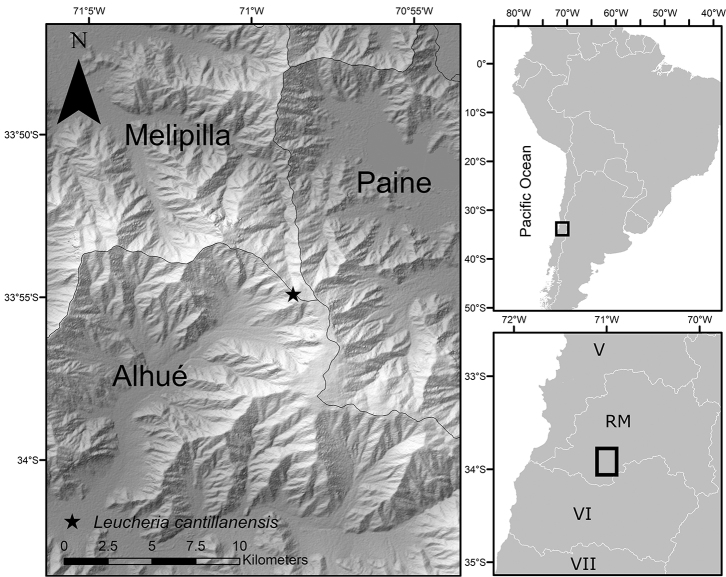
Distribution map of *Leucheria cantillanensis* (star) in Chile, Región Metropolitana, based on the type locality.

During the austral summer of 2019, a botanical exploration was made to the coastal mountain range of the Metropolitan Region of Chile, specifically to the Reserva Natural Altos de Cantillana (Fig. 1). Specimens of *Leucheria* that could not be assigned to any of the accepted species for the genus were found. Herbarium specimens were collected, together with leaf material preserved in silica gel, as well as material preserved in alcohol 70%. Herbarium specimens were deposited to CONC and SGO herbaria. A systematic examination of herbarium specimens of *Leucheria* found at CONC and SGO, as well as online digital images of specimens available on E, K, and P, was carried out. The descriptions and keys were prepared after examining all available specimens. Terminology for describing floral parts follows Simpson (2010) and Beentje (2016).

### Conservation status

The assessment of the conservation status of the species was made using the International Union for Conservation of Nature (IUCN 2017) criteria. The extent of occurrence (EOO) and area of occupancy (AOO) were calculated using GeoCat (Bachman et al. 2011).

### Taxon sampling for phylogenetic analysis

DNA sequences for nDNA (ITS), as well as cpDNA intergenic spacers (*rpl32-trnL* and *trnF-trnL*) were obtained from GenBank (www.ncbi.nlm.nih.gov/Genbank) for all species of *Leucheria* recognized in the phylogenetic reconstruction of the genus (Jara-Arancio et al. 2017). Sequences for the putative new species were generated in the present study. As outgroups, we used sister taxa of *Leucheria* from Nassauvieae tribe: *Moscharia pinnatifida* Ruiz & Pav., *Moscharia solbrigii* Crisci, *Marticorenia foliosa* (Phil.) Crisci and *Spinoliva ilicifolia* (Hook et. Arn.) G. Sancho.

### DNA extraction, amplification, sequencing, and phylogenetic analyses

Total genomic DNA was extracted from silica-dried material collected in the field from the type specimen using the Qiagen DNeasy Plant Mini Kit (QIAGEN, Santiago, Chile) following the manufacturer’s instructions. Genomic DNA was used to amplify by PCR the internal transcribed spacer region (ITS) and the chloroplast *trnL-trnF* (Taberlet et al. 1991) and *rpl32-trnL* (Shaw et al. 2007) intergenic spacers. We amplified all regions in 25 μl PCR reactions following thermocycling procedures used in Jara-Arancio et al. (2017). Sanger sequencing was performed in the Plataforma de Secuenciación y Tecnologías Ómicas, Pontificia Universidad Católica de Chile, using the ABI PRISM 3500 xl Genetic Analyzer (Applied Biosystems). GenBank accession numbers for all DNA sequences are given in Suppl. material 1.

The assembled sequences were aligned using the ClustalW algorithm in Geneious Prime 2019.1.1 (https://www.geneious.com). Phylogenetic analyses were carried for both Maximum-likelihood (ML, Felsenstein 1981), using RAxML-AVX3 version (Stamatakis 2014) included in RAxMLGUI v.2.0 beta (Silvestro and Michalak 2012, Edler et al. 2019) and Bayesian inference (BI) using MrBayes x64 v3.2.7 (Ronquist et al. 2012) respectively. The best-supported model of nucleotide sequence evolution for each partition was determined based on the Akaike Information Criterion (AIC) using MrModeltest v2 (Nylander 2004). For the combined analysis with BI, three partitions were used corresponding for each region, in which evolutionary models for each one were: SYM+I+G in ITS; GTR+G in *rpl32-trnL* and GTR+I+G in *trnL-trnF*. Maximum likelihood analyses were run using the GTRGAMMA approximation, which approximates to a GTR model. Substitutions parameters were estimated independently for the nuclear and plastid partitions. The analysis included 1000 ML slow bootstrap replicates with 100 runs. Bayesian analyses were conducted under the respective best fit models for each partition, with two independent runs for 15 million generations, sampling every 10000 generations. Time series plots and effective sample size (ESS) were analyzed using TRACER v.1.7 (Rambaut et al. 2018) in order to check convergence for each run. The first 3 million generations were discarded as burn-in.

## Results

### Molecular phylogenetic analyses

**Figure 2.**
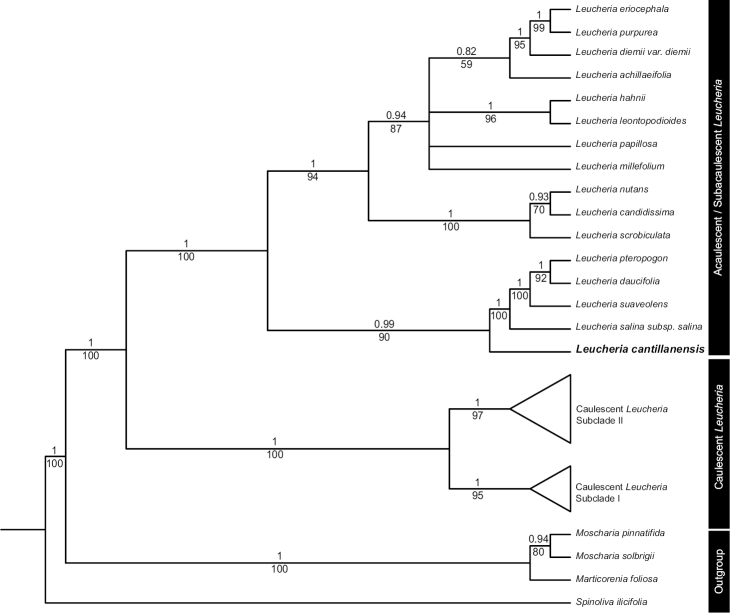
Phylogeny of *Leucheria* resulting from Bayesian analysis of the combined nuclear ITS and plastid *rpl32-trnL* and *trnL-trnF* dataset. Numbers above and below the branches represent the Posterior probabilities from the BI analysis and bootstrap values from the ML analysis, respectively. Nodes with <0.5 PP were collapsed to polytomies. The new species, *Leucheria cantillanensis* is highlighted in bold.

The DNA matrix contained 2962 nucleotide characters (782 ITS, 1224 *rpl32-trnL* and 956 *trnL-trnF*), representing 44 ingroup and 4 outgroup accessions. BI and ML analyses yielded congruent topologies. The topology of the phylogenetic tree constructed in this study is congruent with the three major clades within the genus found by Jara-Arancio et al. (2017), with slight differences at lower resolution (Fig. 2). Both caulescent clades I and II formed a well-supported clade (BS=97, PP=1.0; BS=95, PP=1.0, respectively). The acaulescent/subacaulescent clade formed a well-supported clade (BS=90, PP=0.99). *Leucheria cantillanensis* is nested within the latter clade, being sister taxa to Leucheria salina (J. Remy) Hieronymus subsp. salina, *Leucheria suaveolens* (D’Urv.) Spegazzini, *Leucheria pteropogon* (Griseb.) Cabrera and *Leucheria daucifolia* (Don) Crisci.

### Taxonomic treatment

#### Leucheria cantillanensis

Taxon classificationPlantaeAsteralesAsteraceae

Lavandero sp. nov.

C2CD131A-1BBA-5302-8CC7-F0723D7790DF

urn:lsid:ipni.org:names:77213224-1

Figures 3 , 4

##### Diagnosis.

*Leucheria cantillanensis* is similar to *L. salina* but differs in its flat lamina and chartaceous texture (vs. foliar segments perpendicular or oblique to the lamina axis and leathery texture) (Figs 5C, D, 6G, I), conspicuously prominent venation, with the secondary and tertiary veins forming a raised pattern above and below the lamina (vs. non-prominent venation, with no raised pattern of veins in either side of the lamina) (Fig. 6G, I), 27–30 flowers per capitula (vs. 40–60), purple anther apical appendages (vs. greenish-yellow) (Fig. 6D, F). *Leucheria cantillanensis* is also similar to coastal forms of *L. runcinata* D.Don with white corollas, but it differs by its underground creeping rhizome with nodes and leaf scars and remnants of dry leaf petioles along the rhizome (vs. a lignified taproot with leaf scars concentrated at the base of the stem at the surface of the ground) (Fig. 6K, L), entirely glandular indumentum in the leaves (vs. glandular on the adaxial side and glandular and lanate on the abaxial side) (Fig. 6H, I), conspicuous prominent venation on both sides of the lamina (vs. inconspicuous and only midvein prominent on the abaxial side) and purple anther apical appendages (vs. greyish-blue) (Fig. 6E, F).

##### Type.

Chile. Región Metropolitana: Provincia de Melipilla, entre el límite de Alhué y Melipilla, Reserva Natural Altos de Cantillana, 33°54'54.24"S, 70°58'43.57"W, 2007 m., 27 December 2019, fl. And fr., *Lavandero* 700 (holotype: CONC!; Isotype SGO!).

##### Description.

***Perennial*** caulescent herb 15–30(–40) cm tall, decumbent, forming clumps of 5–6 aerial stems arising from the apex and nodes of the distal end of the rhizome. ***Rhizome*** dark brown, round, 10–15 mm wide, oblique to creeping, leafless below, but with remnants of dry leaf petioles, roots arising from the internodes. ***Roots*** dark brown, ca. 2 mm wide, round in cross-section. ***Stems*** purplish at the base, green at the top, 1.0–5.5 mm wide, simple or branching, round in cross-section, internodes up to 4 cm long, densely covered by glandular, capitate, (87–)115–180 µm long, multicellular (6–12-celled) trichomes with clear resin, fragrant, with pungent citric scent (same indumentum up to the corolla tube). ***Leaves*** dark green, alternate; basal leaves petiolate, semi-densely arranged at the base; petiole compressed, winged, vaginate, 2–2.5(–3.5) cm long; upper leaves sessile, amplexicaul, loosely arranged, gradually reduced in size towards the capitulescences. ***Lamina*** obovate, (10-)50–100(–140) × (5–)20–25(–35) mm; base attenuate, amplexicaul, apex mucronate; margin serrate, texture chartaceous, densely glandulous on both surfaces; pinnatisect to entire towards the tip; segments at the base entire, rarely 1(–2)-dentate, apex mucronate; segments in the middle (3–)4–6(–7)-dentate; apical segments fused, doubly dentate; venation conspicuously prominent, with the secondary and tertiary veins forming a raised pattern on both sides of the lamina, pinnate, semicraspedodromous, with primary vein ending in apical mucro, secondary veins either ending in second-order teeth or joining other distal secondary veins. ***Capitulescences*** a corymbiform cyme. ***Capitula*** 1–6 per stem, pedunculate, (0.5–)1.2–2.8(–3.6) cm long, homogamous, discoid. ***Involucres*** hemispheric 7.0–7.2 × 8.2–8.3 mm, two-seriate, alternate. A third series of Middle involucral bracts with intermediate characters between outer and inner Middle involucral bracts rarely present. ***Receptacle*** slightly convex, epaleate, glabrous. ***Outer Middle involucral bracts*** 5–6, green, lanceolate, concave on the inner face, 6.6–7.6 × 1.10–1.21(–1.36) mm, with 3 dark-green longitudinal stripes (including the midrib), apex ciliate, margin entire, texture leaf-like, abaxial lamina and margins densely covered by glandular trichomes, adaxial lamina glabrous. ***Middle involucral bracts*** rarely present, 1–2, green, lanceolate, concave to flat, with 6.5–7.0 × 1.08–1.19 mm, with 3 dark-green longitudinal stripes (including the midrib), apex ciliate, texture leaf-like to hyaline towards the margins, margin ciliate, rarely glandular, central portion of the abaxial lamina covered by glandular trichomes, hyaline lamina glabrous. ***Inner Middle involucral bracts*** 5–6(–8), green, lanceolate, concave to flat 6.2–7.1 × 1.06–1.25(–1.51) mm, with 3 dark-green longitudinal stripes (including the midrib), apex acute, texture leaf-like to hyaline-membranaceous towards both lateral margins, margin ciliate, cilia (0.11–)0.17–0.21(–0.24) mm long, central portion of the abaxial lamina densely covered by glandular trichomes, hyaline lamina glabrous, adaxial lamina glabrous. ***Flowers*** isomorphic, bisexual, 27–30 per capitulum. ***Corollas*** bilabiate, white, before anthesis pinkish-white, tube 3.7–4.0 mm long, 0.5–1.1 wide; corolla tube sparsely covered by glandular trichomes. ***Outer lip*** oblanceolate, 3.6–3.9 × 2.0–2.2 mm at its widest, apex 3-toothed, teeth equal, 4-veined, glabrous. ***Inner lip*** bifid, lacinae linear, 2.7–2.8 × 0.24–0.37 mm at its widest, connivent, glabrous. ***Stamens*** 5, 3.8–4.0 mm long, glabrous. ***Anthers*** sagittate, 3.25–3.29 mm long; apical appendages purple, lanceolate, 1.19–1.23 mm long, apex acute; tails long, lanceolate, ca. 0.6 mm long, apex acute, smooth to ciliate. ***Styles*** white, 4.2–5.0 mm long, cleft into two truncate branches, branches 0.54–0.73 mm long, with stigmatic papillae on internal surface. ***Cypselae*** dark-brown, 1.0–1.2 × 2.4–2.5 mm, obovoid, strigose; trichomes transparent, cylindric, terete, (150–)167–170(–180) µm, ascending, unicellular, subtended by two globose exocarpic cells. ***Pappus*** uniseriate, fused at their bases into a ring, deciduous; bristles 19–20, white, capillary, sub-plumose, 4.5–5 mm long; pectines long, filiform, 0.21–0.35(–0.46) mm long, laterally inserted.

**Figure 3.**
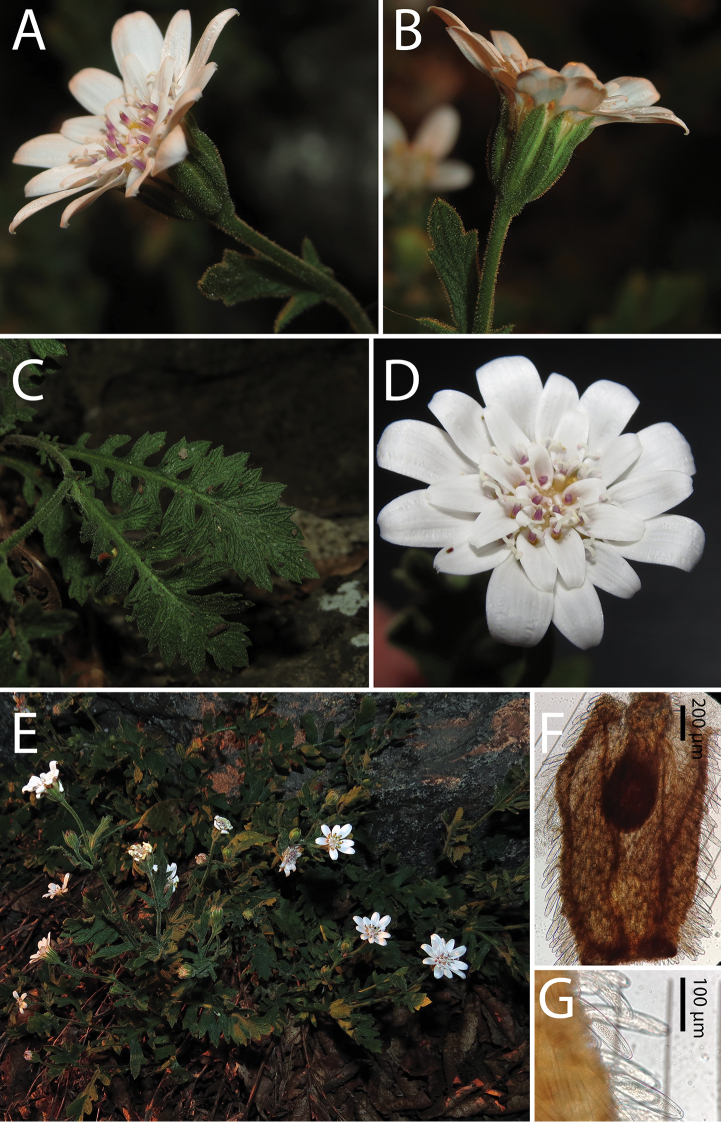
*Leucheria cantillanensis* Lavandero, sp. nov. **A** capitulum, sideways view **B** capitulum, detail of Middle involucral bracts **C** leaves **D** capitulum, upper view **E** plants growing in natural habitat **F** cypsela **G** detail of trichomes in cypsela. All photographs by Nicolás Lavandero.

**Figure 4.**
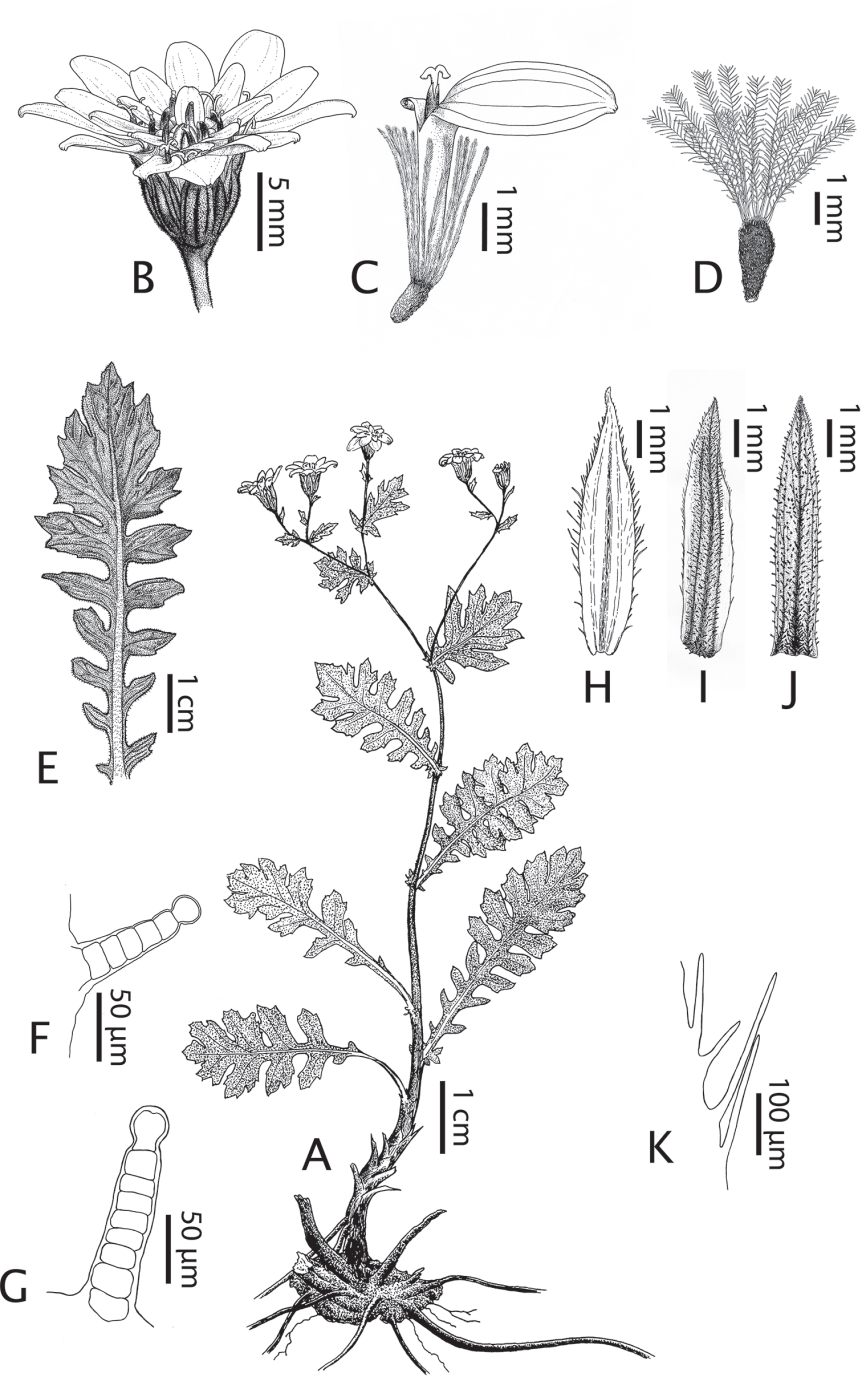
*Leucheria cantillanensis* Lavandero, sp. nov. **A** habit **B** capitulum **C** detail of flower **D** fruit **E** basal leaf **F** leaf trichome **G** stem trichome **H** inner Middle involucral bract **I** middle Middle involucral bract **J** outer Middle involucral bract **K** ciliate margin of inner Middle involucral bract (detail). Illustrations by Benito Rosende.

##### Distribution and habitat.

*Leucheria cantillanensis* seems to be endemic to the Cantillana Mountain Range, which is part of the coastal mountain range of central Chile. It grows in shaded crevices of rocky outcrops near 2000 m a.s.l. with SW orientation (Fig. 7). It is known thus far only from the type locality (Fig. 1). *L. cantillanensis* occurs associated with other rupicolous taxa such as *Calceolaria andina* Benth.

##### Phenology.

Collected flowering and fruiting in December.

##### Etymology.

The specific epithet refers to the coastal mountain range where the species was found, Altos de Cantillana.

**Figure 5.**
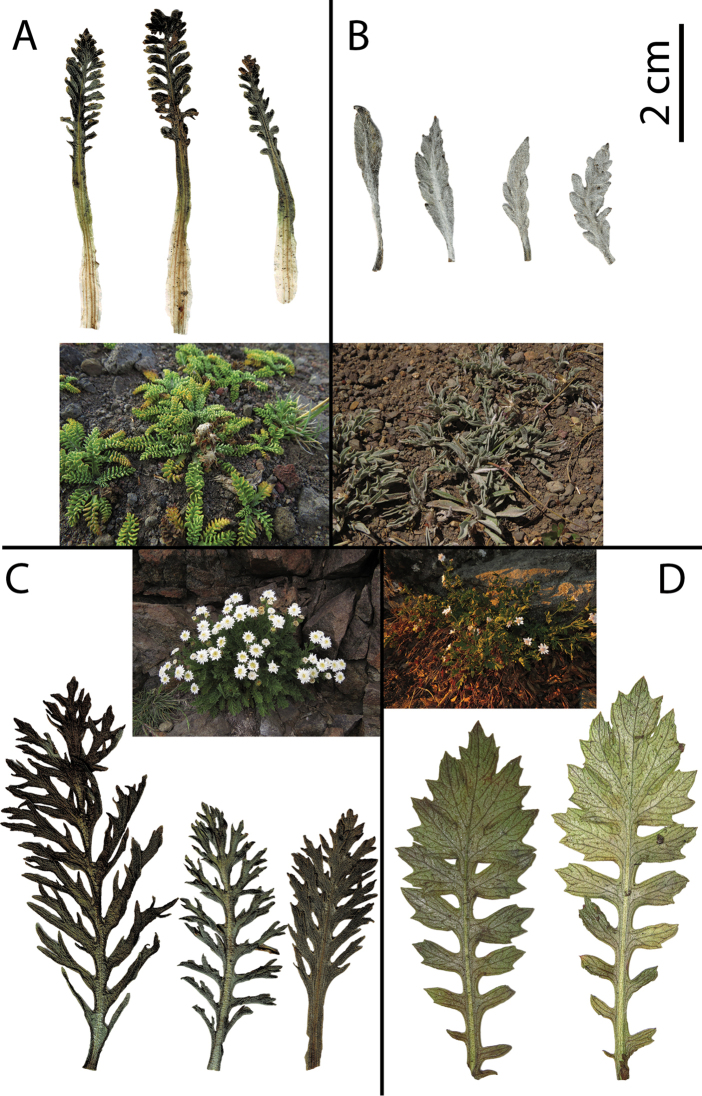
Leaf morphology and habit of acaulescent/subacaulescent *Leucheria* from Central Chile **A***Leucheria scrobiculata* (NL433, CONC), inset: Habit **B***Leucheria candidissima* (Lavandero, Santilli & Ossa 36, CONC), inset: Habit **C***Leucheria salina* (Lavandero & Abello 40, CONC), inset: Habit **D***Leucheria cantillanensis* (NL700, CONC), inset: Habit. All photographs by Lavandero.

##### Conservation status.

*Leucheria cantillanensis* is assessed here as Critically Endangered (CR) under the IUCN categories and criteria B2ab(i,ii,iii). Criterion B2 was selected because its Area of Occupancy is <10 km^2^ (4 km^2^). Criterion “a” was selected because it is known to exist at only a single location, with only one subpopulation. Criterion b(i,ii,iii) was selected because we expect a continuing decline of suitable area for the species to exist in since it is only found at the highest elevations within the mountain coastal range with very specific soil types and exposition. The quality of its habitat has also been deteriorating over time. The overall precipitation and snow cover in the Cantillana plateau has decreased dramatically over the past 20 years, affecting not only the Andean relict flora but all the vegetation in the area. Fog events which compensate for the drought over the summer, are not as common as before. *Leucheria cantillanensis* is present in the private reserve “Reserva Natural Altos de Cantillana”.

**Figure 6.**
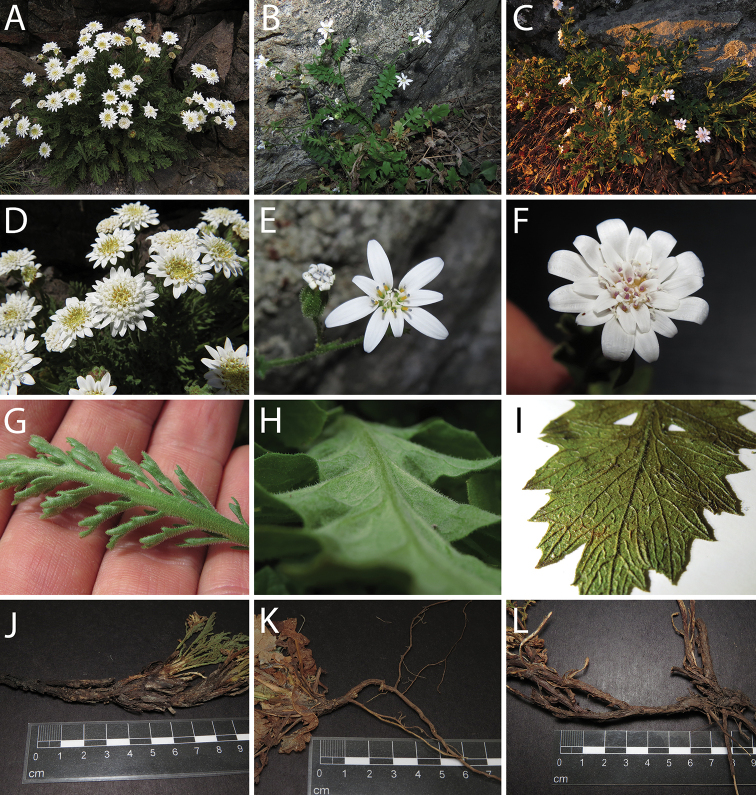
*Leucheria salina* (**A, D, G, J**) (Valle Nevado, Región Metropolitana, Chile), *Lecheria runcinata* (**B, E, H, K**) (Reserva Natural Altos de Cantillana, Región Metropolitana, Chile), and *Leucheria cantillanensis* (**C, F, I, L**) (Reserva Natural Altos de Cantillana, Región Metropolitana, Chile) **A–C** habit **D–F** capitula **G–I** abaxial side of leaves **J–L** belowground structures. All Photographs by Lavandero.

**Figure 7.**
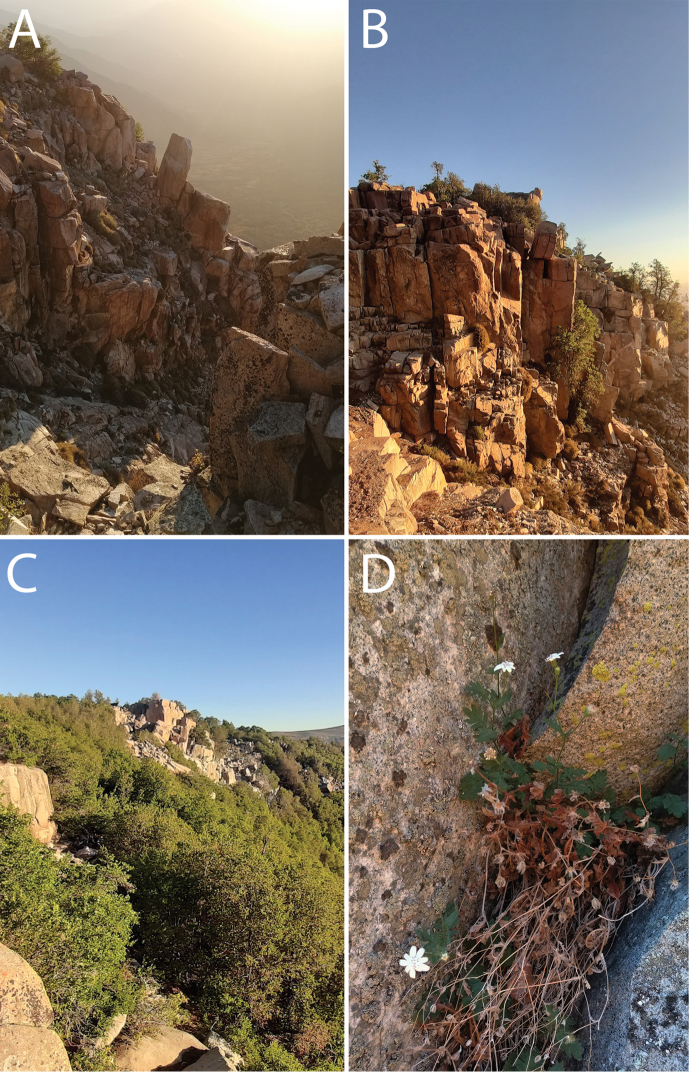
Habitat of *Leucheria cantillanensis* in Central Chile **A** southwest facing rock outcrops, ca. 2000 m elevation **B** rock outcrops in Cantillana, general view **C** general view of rock outcrops among *Nothofagus macrocarpa* (A.DC.) F.M. Vázquez & R.A. Rodr. forests in the Cantillana plateau **D***Leucheria cantillanensis*, detail of the plant growing between rock crevices. Photographs **A, D** by Lavandero, **B, C** by Fabiola Gamboa.

### Key to acaulescent/subacaulescent species of *Leucheria* of Central Chile (31°–34°S)

The caulescent herbs with lignified taproots and annual species are excluded. It is important to observe the belowground structures in fresh and dry specimens in order to correctly assign the species to this group. For the habit, please refer to the insets in Figure 5.

**Table:** 

1	Plants densely tomentose, greyish; Corolla pink	***L. candidissima***
–	Plants not densely tomentose, glabrous or glandulous; Corolla white to bluish	**2**
2	Plants caespitose, up to 8 cm tall, glabrous to glabrescent	***L. scrobiculata***
–	Plants erect or decumbent, taller than 8 cm, densely glandulous	**3**
3	Lamina with foliar segments perpendicular or oblique to the axis of the lamina, non-prominent venation; flowers 40–60 per capitula, anther apical appendages greenish-yellow; Andes of Chile and Argentina above 3000 m.a.s.l.	***L. salina***
–	Lamina flat with foliar segments on the same axis of the lamina, conspicuously prominent venation; flowers 27–30 per capitula; anther apical appendages purple. Coastal Cordillera of Chile near 2000 m.a.s.l.	***Leucheria cantillanensis***

## Discussion

The ability to establish infrageneric relationships using classic chloroplast markers and ITS within *Leucheria* is demonstrated in the present study. We were able to corroborate our initial conjectures about the phylogenetic position of *Leucheria cantillanensis* within the genus. Based on belowground structures (rhizome and roots), we assumed this species belonged to the acaulescent/subacaulescent group found by Jara-Arancio et al. (2017). This group is characterized by plants with a basal and compact rosette, with one or few monocephalic (sometimes with more capitula) scapes. In the first phylogenetic study with molecular data, Jara-Arancio et al. (2017) found that the independent acaulescent evolutionary lines (*L. candidissima* and *L. salina* lines) proposed by Crisci (1976) were paraphyletic, as they should include the species *L. achillaeifolia* and *L. nutans* in order to be monophyletic. We believe that this finding is highly supported by morphologic characters, as all the species within this clade have the same belowground structures, such as horizontal rhizomes covered by old leaves, and rounded in cross-section, long, dark roots. These characters are distinctly different from the other clades found by Jara-Arancio et al. (2017), which can be either annual or perennial plants with a lignified taproot. *Leucheria cantillanensis* evidently belongs to the acaulescent/subacaulescent group, as it has a creeping rhizome that grows between rock crevices and long dark roots (Figs 4, 6). Among the genus *Leucheria*, *L. cantillanensis* can be easily distinguished by its unique combination of vegetative (Figs 5, 6) and sexual characters (Figs 3, 4, 6), as described in detail in the diagnosis.

From the biogeographic point of view, it is interesting to note that the closest species of *L. cantillanensis* are mainly found at high elevations in the Andes range (except for *L. suaveolens*, which is endemic to the Falkland Islands). Although we do not have a time-calibrated phylogeny for *Leucheria*, it is possible to hypothesize, based on Villagrán (2001) and Villagrán and Armesto (2005), that the species diverged from this mainly Andean clade during the repeated glacial/interglacial cycles during the Quaternary. Cold-adapted species occupied lower elevation during glacial periods, and a subsequent shift upwards towards high elevations in both Andean and coastal mountain ranges during warmer periods likely provided the opportunity for allopatric speciation.

*Leucheria cantillanensis* is an exclusively rupicolous species. Plant communities on rocky places are characterized by having high levels of endemism caused by the high specialization that plants require to thrive in these specific habitats (Porembski et al. 1994; Larson et al. 2005). Currently, only one population of *L. cantillanensis* is known. The area of Cantillana mountain range with elevations >2000 m comprises an area of near 15 km^2^ (EULA-Chile 2004) but the specific rocky outcrops and exposition where the species is found, dramatically reduces the suitable habitat for the species. What is more, during the last decade, Central Chile has experienced a severe deficit in precipitation, the so-called Mega Drought (Garreaud et al. 2020), which has caused major damage and changes to the vegetation in the area (Miranda et al. 2020). Observations by the Park rangers over the past years reveal a decrease in snow cover in the mountain plateau and a reduction of fog and cloud events, an important source of water for rupicolous taxa. In this scenario, it is possible to infer a continuous decline in the quality of its habitat and a projected decline of suitable habitat and population size in the near future. *Ex situ* conservation measures could be a cost-efficient method to preserve the species, but more information about its distribution, ecology and population size is needed.

The discovery of this new species, which is restricted to mountain tops in the Cantillana Mountain Range in Central Chile, highlights the importance of this site in terms of its unique biodiversity. Cantillana harbours several taxa endemic to the Mediterranean region of Chile (Romero and Teillier 2009, Romero-Gárate and Teillier 2014) and several new species of insects, lizards and amphibians have been described over the recent years (Vaz-de-Mello and Halffter 2006; Núñez 2007; Zúñiga-Reinoso and Cid-Arcos 2013; Charrier et al. 2015) in the area. Rock communities have been rarely studied in Chile (García 2010). Further botanical surveys in rocky outcrops may reveal a hidden diversity of plants in this biodiversity hotspot and will provide important information needed for the management and conservation of *L. cantillanensis*.

### Additional specimens examined

*Leucheria salina*. **CHILE. Atacama**: Huasco, Quebrada Cantarito, 28°39'S, 69°50'W, Feb 1981, Kalin 81614 (CONC); **Coquimbo**: Limarí, Cordillera de Ovalle, Río Mostazal, 30°46'S, 70°30'W, Feb 1956, Jiles 2971 (CONC); Cordillera de Ovalle, San Miguel, 30°51'S, 70°31'W, Jan 1954, Jiles 3638 (CONC); Gordito, 31°04'S, 70°23'W, Jan 1954, Jiles 2546 (CONC); **Valparaíso**: Los Andes, entre estero Caracoles y Cristo Redentor, 34°00'S, 70°06'W, Apr 1933, Looser 67007 (CONC), Caracoles, 32°50'S, 70°07'W, Jan 1964, Marticorena & Matthei (CONC); Portillo 32°50'S, 70°08'W, Feb 1951, Ricardi 1207 (CONC); **Región Metropolitana**: Santiago, Cordillera Santiago ad limit nivis perpet, Feb 1854, Philippi s/n (SGO); SN Yerba Loca, Cuenca Estero La Leonera, 33°16'S, 70°15'W, Feb 2000, Kalin et al. 201451 (CONC); Subida al Portezuelo de El Cepo, 33°18'S, 70°12'W, Mar 1956, Schlegel 1087 (CONC); Farellones, La Parva, 33°19'S, 70°16'W, Jan 1991, Ruthsatz 6995; Farellones, Laguna Piuquenes, 33°19'S, 70°15'W, Feb 2019, Lavandero & Abello 1890 (SGO); Cerca de la Parva, 33°18'S, 70°17'W, Jan 1979, Muñoz & Meza 1372 (SGO); Río Colorado, 33°28'S, 70°00'W, Jan 1930, Behn s/n (CONC); San Ramón, 33°28'S, 70°25'W, Jan 1967, Zoellner 1430 (CONC); El Volcán, 33°48'S, 70°10'W, Feb 1947, Gunckel 20683 (CONC); Maipo, Cajón del Maipo, 33°48'S, 70°44'W, Jan 1980, Niemeyer s/n (CONC); Santiago, Parque Nacional El Morado, 33°49'S, 70°05'W, Jan 1991, Teillier et al. 2402 (CONC, SGO); Cercanías glaciar La Paloma, Feb 2014, Medina 2616 (SGO).

*Leucheria runcinata*. **CHILE. Valparaíso**: Nogales, Cordillera El Melón, Estero Garretón 32°39'S, 71°02'W, Nov 2010, Flores-Toro s/n (SANT); Quillota, Cerro La Campana, sector La Gotera, 32°58'S, 71°08'W, Jan 1937, Garaventa 3253 (CONC); Cerro La Campana, 32°57'S, 71°08'W, Nov 1962, Weisser 395 (CONC); Cerro La Campana, Trayecto Placa Darwin a Mina, 32°57'S, 71°08'W, Dec 1981, Villagrán & Meza 3174 (SGO); Quillota, Cerro Vizcachas, 33°05'S, 71°02'W, Nov 1973, Stebbins 8912 (SGO); **Región Metropolitana**: Chacabuco, Altos de Chicauma, sector Tranque, 33°10'S, 70°58'W, Jan 2003, García & Faúndez 3637 (CONC); Melipilla, Reserva Natural Altos de Cantillana, sendero camino a cerro Horcón de Piedra, 33°53'S, 71°00'W, 27 Dec 2019, Lavandero 753 (SGO).

## Supplementary Material

XML Treatment for Leucheria cantillanensis

## Supplementary materials

Supplementary material 1 Table S1. GenBank accession numbers used in this study Data type molecular data Explanation note GenBank accession numbers for the ITS, trnL-trnF and rpl32-trnF sequences used in this study. GenBank accessions in bold are new to this study. https://binary.pensoft.net/file/484187This dataset is made available under the Open Database License (http://opendatacommons.org/licenses/odbl/1.0/). The Open Database License (ODbL) is a license agreement intended to allow users to freely share, modify, and use this Dataset while maintaining this same freedom for others, provided that the original source and author(s) are credited.Nicolás Lavandero
